# Whole genome sequencing to investigate a putative outbreak of the virulent community-associated methicillin-resistant *Staphylococcus aureus* ST93 clone in a remote Indigenous community

**DOI:** 10.1099/mgen.0.000098

**Published:** 2016-12-12

**Authors:** Ella M. Meumann, Patiyan Andersson, Fiona Yeaman, Sarah Oldfield, Rachael Lilliebridge, Stephen D. Bentley, Vicki Krause, Miles Beaman, Bart J. Currie, Deborah C. Holt, Philip M. Giffard, Steven Y. C. Tong

**Affiliations:** ^1^​ Global and Tropical Health Division, Menzies School of Health Research, Charles Darwin University, Darwin, Northern Territory, Australia; ^2^​ Department of Infectious Diseases, Royal Darwin Hospital, Darwin, Northern Territory, Australia; ^3^​ Centre for Disease Control, Department of Health, Northern Territory Government, Darwin, Northern Territory, Australia; ^4^​ Wellcome Trust Sanger Institute, Hinxton, Cambridge, UK; ^5^​ School of Pathology and Laboratory Medicine, University of Western Australia, Perth, Western Australia, Australia

**Keywords:** MRSA outbreak Indigenous whole genome sequencing

## Abstract

We report two cases of severe pneumonia due to clone ST93 methicillin-resistant *Staphylococcus aureus* (MRSA) presenting from a remote Australian Indigenous community within a 2-week period, and the utilization of whole genome sequences to determine whether these were part of an outbreak. *S. aureus* was isolated from 12 of 92 nasal swabs collected from 25 community households (including the two index households); one isolate was ST93. Three of five skin lesion *S. aureus* isolates obtained at the community were ST93. Whole genome sequencing of the ST93 isolates from this study and a further 20 ST93 isolates from the same region suggested that recent transmission and progression to disease had not taken place. The proximity in time and space of the two severe pneumonia cases is probably a reflection of the high burden of disease due to ST93 MRSA in this population where skin infections and household crowding are common.

## Data Summary

1. Short read data for the six community ST93 MRSA isolates are available at the SRA; Bioproject PRJNA286158 (http://www.ncbi.nlm.nih.gov/bioproject/PRJNA286158/)
2. Short read data for the additional 20 NT ST93 isolates are available at the ENA; Study PRJEB3144 (http://www.ebi.ac.uk/ena/data/view/PRJEB3144)

## Impact Statement

There is a high burden of severe infection due to methicillin-resistant *Staphylococcus aureus* (MRSA) in remote Australian Indigenous communities, where overcrowding is common. We report two cases of severe infection due to this bacterium from the same small community within a 2-week period, which raised concern that there could be an outbreak. We undertook a contact tracing exercise and used whole genome sequencing to investigate the relatedness of MRSA isolates. Our results suggested that an outbreak was not taking place, and provided insight into the complexity of MRSA transmission in the Northern Territory of Australia.

## Introduction

*Staphylococcus aureus* is a common cause of community-acquired sepsis in Indigenous populations (Tong *et al.*, 2009, 2012), and the incidence of community-associated (CA) methicillin-resistant *S. aureus* (MRSA) has increased (Tong *et al.*, 2015b). Despite the heavy burden of infectious diseases in Indigenous populations, investigation of transmission and putative outbreaks in Indigenous communities using whole genome sequencing has been rare to date. Typically, whole genome sequencing studies for outbreak investigations of MRSA have occurred in the hospital setting (Koser *et al.*, 2012; Harris *et al.*, 2013; Tong *et al.*, 2015a). Here, we provide an example of an investigation of a putative outbreak of a virulent CA-MRSA clone in a remote Indigenous community.

In Australia, sequence type (ST)93 MRSA has emerged as the dominant CA-MRSA strain (Coombs *et al.*, 2014a, b). ST93 is a highly virulent *S. aureus* clone that is associated with necrotizing pneumonia (Peleg & Munckhof, 2004; Peleg *et al.*, 2005; Risson *et al.*, 2007; Tong *et al.*, 2008), severe skin and soft tissue infection (SSTI), and bone and joint infections (Nourse *et al.*, 2007; Tong *et al.*, 2010). Invasive infection due to ST93 *S. aureus* often affects young people (Peleg *et al.*, 2005; Nourse *et al.*, 2007), and is usually severe and sometimes fatal (Peleg & Munckhof, 2004; Risson *et al.*, 2007; Tong *et al.*, 2008).

During a 2-week period in August 2013, two young Indigenous patients from the same remote community were evacuated to Royal Darwin Hospital with severe pneumonia due to ST93 MRSA. The remote community is located ~500 km from the main city of Darwin and has a population of approximately 1300 people. This raised concern that there could be an outbreak of this virulent strain. Here we describe the contact tracing exercise that was undertaken and the comparison of genome sequences of *S. aureus* ST93 MRSA isolates from the community and the region.

## Methods

The purpose of the contact tracing exercise was explained to community members with the aid of a flip chart and a local interpreter where required. Written informed consent was obtained from each study participant or their guardian. The investigation was initially registered as a quality assurance audit (QAAR 2013-2107), and subsequently a complete research ethics application was approved (HREC 2015-2368) by the Human Research Ethics Committee of the Northern Territory Department of Health and Menzies School of Health Research.

During a 2-week period in September 2013, we visited the households of the two index cases. All members of the two households had nasal, throat and groin swabs collected, and skin lesions swabbed if present. Additionally, we collected nasal swabs from a sample of patients who had presented to the community health clinic, and we also obtained *S. aureus* isolates from patients with SSTI at this time.

The swabs were collected and transported as previously described (Bowen *et al.*, 2014). We extracted DNA from *S. aureus* isolates using a QiaAMP DNA Mini Kit (Qiagen) according to the manufacturer’s instructions. Genotyping to the level of clonal complex was by a high-resolution melting analysis method previously described (Lilliebridge *et al.*, 2011). In addition, PCR assays were used to detect the *nuc* (Brakstad *et al.*, 1992), *mecA* (Hiramatsu *et al.*, 1992) and *lukSF-PV* (Stephens *et al.*, 2006) genes. Whole genome sequencing of ST93 isolates was carried out on an Illumina HiSeq platform by Macrogen or through a collaborative agreement with the Wellcome Trust Sanger Institute. The ST93 genomes already published provided a phylogenetic framework for the analysis (Stinear *et al.*, 2014) and we sequenced an additional 20 ST93 isolates obtained between 2005 and 2010 from Northern Territory (NT) hospital-based studies to provide local genomic context (Tong *et al.*, 2009; Brennan *et al.*, 2013).

We aligned sequence data from existing isolates (Stinear *et al.*, 2014) and newly generated sequences against the JKD6159 reference genome (ST93, GenBank: CP002114), using spandx v2.6, with default parameters to produce a core alignment (Sarovich & Price, 2014). Orthologous SNPs were used for phylogenetic reconstruction using RAxML (Stamatakis, 2014). Mobile genetic elements were not excluded.

## Results

### The cases

The two cases were a 9-year-old Indigenous male and a 24-year-old Indigenous female from the same remote Indigenous community who presented to the community clinic on 16 August and 29 August 2013, respectively. Both developed septic shock and respiratory failure, and required transfer to the Royal Darwin Hospital Intensive Care Unit for ventilatory and inotropic support. Both patients were diagnosed with CA pneumonia and ST93 MRSA was isolated from sputum or blood culture samples.

### Outbreak investigation

Nose, throat and groin swabs were obtained from six and nine members of the two index case households. None of these individuals had skin lesions. Nose swabs were also obtained from 77 people from 23 other households in the same community. Three of these individuals also had skin lesions which were also swabbed. In total, 92 individuals had nasal swabs collected; the median age was 26 years (interquartile range 9–41), 59 (64 %) were over 18 years of age and 64 (70 %) were female.

From the 92 nasal swabs, *S. aureus* was cultured from 12 individuals (13 %), including one swab collected from a member of index household 1. *S. aureus* was not isolated from any of the throat or groin swabs. Two of the three swabs collected from skin lesions had *S. aureus* isolated. A further three *S. aureus* isolates were recovered from three patients who had presented to the community health clinic with skin infections. The results of the genotyping of these *S. aureus* isolates are presented in Table 1. Three of five (60 %) *S. aureus* isolates from skin were ST93 MRSA, while only one of 12 (8 %) nasal *S. aureus* isolates was ST93 (*P*=0.05, Fisher’s exact test). ST93 was not isolated from any members of the index households.

**Table 1.** Source and ST of *S. aureus* isolates

Age (years) and sex	Source	Household	*mecA* result	Clonal complex or ST	PVL result
9M*	Sputum	House 1	Positive	93	Positive
24F†	Blood	House 2	Positive	93	Positive
5M	Skin swab	Other	Positive	93	Positive
47F	Skin swab	Other	Positive	93	Positive
11M	Skin swab	Other	Positive	93	Positive
26F	Nasal swab	Other	Positive	93	Positive
71F	Nasal swab	Other	Positive	1	Negative
37F	Nasal swab	Other	Negative	1	Negative
5M	Skin swab	Other	Positive	1	Negative
4M	Nasal swab	Other	Negative	5	Negative
10F	Nasal swab	Other	Negative	5	Negative
2F	Skin swab	Other	Positive	5	Positive
24F	Nasal swab	Index house 1	Negative	15	Negative
34F	Nasal swab	Other	Negative	45	Negative
41F	Nasal swab	Other	Negative	45	Negative
36F	Nasal swab	Other	Negative	45	Negative
32F	Nasal swab	Other	Negative	101	Negative
24M	Nasal swab	Other	Negative	121	Positive
60F	Nasal swab	Other	Negative	121	Positive

PVL, Panton–Valentine leukocidin toxin.

*Case 1.

†Case 2.

### Comparison of genome sequences

**Fig. 1.**
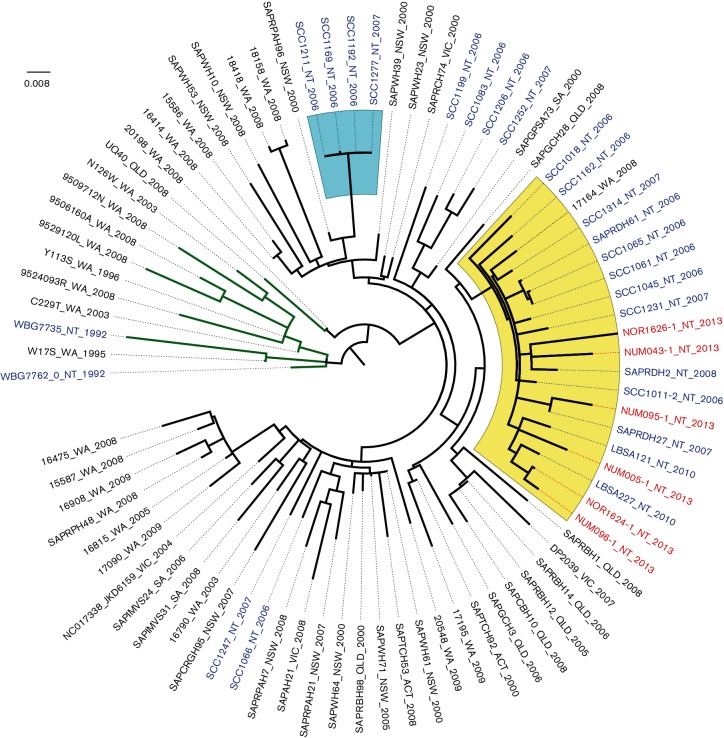
Midpoint rooted, maximum-likelihood phylogenetic tree of ST93 MRSA isolates, including six study isolates, 20 additional NT isolates from our collection and isolates previously sequenced by Stinear *et al.* (2014). Red text, isolates from the study community; blue text, other NT isolates; yellow clade, NT clade; blue clade, clonal cluster; green branches, methicillin-susceptible *S. aureus*; black branches, MRSA; scale bar, nucleotide substitutions per site.

**Fig. 2.**
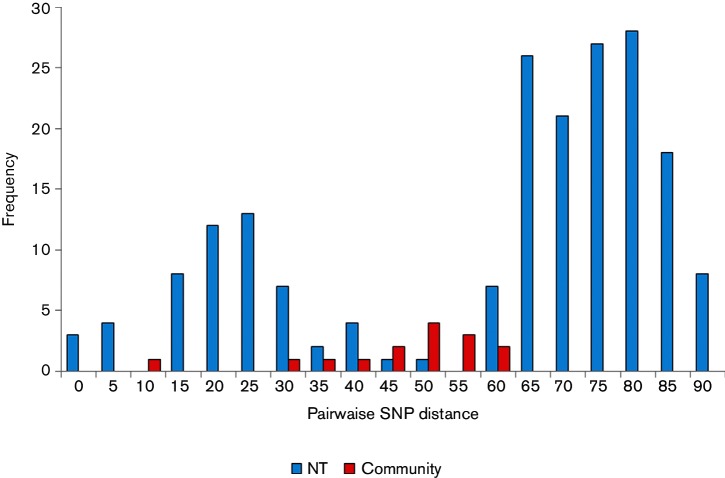
Frequency of pairwise SNP distances for ST93 isolates from the community and the NT.

In addition to the two ST93 MRSA isolates from the index patients, there were four ST93 isolates (three from skin sores and one from a nose swab) identified as part of the contact tracing exercise. The six community study strains, 20 additional NT strains (from hospital-based studies) and all Australian ST93 strains previously sequenced by Stinear *et al.* (2014) were included in a phylogenetic tree (Fig. 1). Details of all included strains are provided in Table S1 (available in the online Supplementary Material). NT isolates were found throughout the tree. The six community isolates were part of a clade made up of predominantly NT isolates; 20 of 21 isolates within this clade were from the NT. Within this clade, the six community isolates were interspersed with other isolates from the NT. The SNP distance between the two index strains was 49. Two isolates were separated by seven SNPs and were from separate households, but we were unable to obtain further epidemiological details to determine if the individuals were otherwise linked. The pairwise SNP differences between the six community strains ranged from seven to 60, with a mean of 44 (Fig. 2). In comparison, the pairwise SNP differences ranged from zero to 88 SNPs (mean 57 SNPs) for all NT strains and from zero to 140 SNPs (mean 66 SNPs) for the entire Australian dataset. We concluded that a recent clonal outbreak was not present because the six community strains were interspersed with other NT strains recovered up to 10 years previously and the distribution of pairwise SNP differences was similar to that of the other NT strains (Fig. 2).

In contrast, there was clear phylogenetic clustering of four of the 20 additional NT isolates that were sequenced (SCC1121, SCC1169, SCC1192, SCC1277). These were separated by zero or one SNP only. We reviewed these cases and determined that the isolates were from three patients in October, November and December 2006. All three were community-onset infections; the patients resided in the Darwin region, and were aged 20, 28 and 39 years. There was no further information available about potential epidemiological links between these individuals.

## Discussion

Whole genome sequencing is the only currently available technique that is able to discriminate adequately between ST93 MRSA isolates in order to understand transmission patterns. Here we provide proof of principal for the use of whole genome sequencing to investigate putative MRSA outbreaks in remote community settings. Although whole genome sequencing has previously been used to investigate household transmission of USA300 MRSA in urban settings in large US cities, the present study provides novel insights from a completely different context.

Alam *et al.* (2015) found that there was a mean of 17.6 (range 0–199) and 12.0 (range 0–102) SNPs separating USA300 isolates within households in Los Angeles and Chicago, respectively. Uhlemann *et al.* (2014) determined that a distance of <23 SNPs was a useful indicator of within-household transmission of USA300. In the UK, Toleman *et al.* (2016) used whole genome sequencing for community *S. aureus* surveillance following a hospital MRSA outbreak; community isolates were separated by 5–20 SNPs from the previous hospital isolates. In our study, the pairwise SNP distance between outbreak investigation isolates was 7–60 SNPs, and between NT isolates was 0–88 SNPs. While the majority of household SNP distances described in the studies above were lower than we identified in our study, there was some overlap, with the SNP distance ranges suggesting that isolates circulating in the remote community and within the NT are closely related.

The six community ST93 MRSA strains were not monophyletic and were interspersed with strains collected up to 10 years previously. This provided convincing evidence that the two putative outbreak isolates did not constitute a discrete lineage within ST93 diversity. In the NT, transmission is probably facilitated by overcrowding of housesholds and there is frequent movement of individuals between households and communities. The phylogeny we describe is reflective of this, and is representative of a broad NT transmission network that has been present for years. Our findings suggest that an outbreak characterized by recent transmission and rapid progression to disease was not taking place, and highlight the importance of interpreting sequence data in the context of other recent local isolates.

Sequencing of the additional NT isolates allowed identification of a contrastingly clonal cluster of four isolates that had been collected from patients in urban Darwin within a 3-month period of 2006. Unfortunately, retrospective epidemiological information was not available for these isolates, and it is likely that they represent part of a previously unidentified outbreak. Whole genome sequencing is likely to play an increasingly important role in public health surveillance of infectious diseases, particularly to prospectively identify outbreaks at an early stage.

In a previous study it was hypothesized that ST93 originated in north-western Australia and subsequently spread to other geographical locations (Stinear *et al.*, 2014). However, only five NT strains were included in that study, and the inclusion of an additional 26 NT strains within the tree has demonstrated greater diversity of NT-derived ST93 isolates than previously appreciated, which is reflective of likely emergence from the tropical north of Australia. As previously described by Stinear *et al.* (2014), our midpoint rooted maximum-likelihood tree demonstrated that methicillin-sensitive *S. aureus* ST93 strains branched earlier and clustered together compared to MRSA strains.

*S. aureus* was isolated only from 12 % of nasal swabs, which may be related to the time taken to transport swabs from a remote setting. The observed pattern of ST93 as a common cause of skin infections but an infrequent nasal colonizer is consistent with other colonization surveys in the NT and Queensland (Vlack *et al.*, 2006; Brennan *et al.*, 2013). In an impetigo treatment trial undertaken in children from NT Indigenous communities, nasal colonization with *S. aureus* was less common among those with *S. aureus*-related impetigo (13 %) compared to those without *S. aureus* impetigo (24 %) (Bowen *et al.*, 2014), suggesting that there may not be a significant overlap of skin and nasal populations of *S. aureus*. This suggests that for some strains, the nose may not be an important reservoir. It is likely that the preferred ecological niches of MRSA are strain-specific (Uhlemann *et al.*, 2014; Knox *et al.*, 2015), and the community reservoirs of ST93 MRSA remain uncertain.

## Conclusion

Whole genome sequencing was used to investigate a potential outbreak of ST93 MRSA. Our results were not consistent with an acute outbreak, but were reflective of a sustained broad NT-wide transmission network. The proximity of the two reported cases in time and space is a reflection of the high burden of disease due to ST93 MRSA infections in Indigenous Australians.

## Supplementary Data

19 Supplementary File 1
